# Beyond compliance: How accreditation can strengthen trust in science and research infrastructure

**DOI:** 10.1017/cts.2026.10241

**Published:** 2026-01-21

**Authors:** Melissa V. Olson

**Affiliations:** Genetic Medicine, The Johns Hopkins University School of Medicine, USA

**Keywords:** research infrastructure, service centers, rigor and reproducibility, quality management, institutional sustainability

## Significance statement

Reproducibility and trust are cornerstones of translational science, yet they are often undermined by weaknesses in the shared resources that support research. This article reframes accreditation, too often seen as a bureaucratic burden, as a strategic tool that strengthens the infrastructure on which discovery depends. By embedding standards, training, and accountability into institutional service centers such as biobanks, genomics cores, and animal research facilities, accreditation enhances rigor, improves efficiency, and builds credibility with funders, collaborators, and the public. Recognizing accreditation as an investment rather than red tape helps institutions improve translational outcomes and sustain competitiveness in an era of tightening budgets and increasing scrutiny.

Science depends on trust, but that trust erodes quickly when results cannot be reproduced or institutional resources fall short of expectations. The so-called reproducibility crisis [1,2] demonstrates that even well-designed experiments can fail when underlying infrastructure is weak–through mishandled samples, poorly calibrated instruments, or inconsistent workflows. Service centers such as genomics cores, imaging facilities, biobanks, metabolomics laboratories, and animal research programs underpin discovery across diverse research areas, yet their role in ensuring rigor and reproducibility often goes unrecognized until failures occur.

Accreditation offers a way to change this narrative. Rather than bureaucratic overhead, it provides a structured framework for accountability, quality, and continuous improvement, transforming service centers from quiet support units into visible drivers of trustworthy and science.

It is important to distinguish accreditation from certification, terms often used interchangeably but representing different concepts. Certification applies to a specific test, process, or individual meeting a defined standard, whereas accreditation evaluates the entire operational system of a laboratory or service center, including governance, quality management, training, documentation, and continuous improvement. Accreditation therefore provides broader assurance of institutional capability and reliability beyond any single test or procedure.

Leading an accredited service center within a large research-intensive medical center provided first-hand insight into both the challenges and benefits of accreditation. The initial phase required substantial effort to formalize policies, processes, and documentation, aligning workflows that had evolved organically. While demanding, accreditation ultimately became a routine maintenance function rather than a recurring burden. Operationally, these changes improved workflows and reduced downstream quality issues. Strategically, accreditation reshaped how the facility was perceived–building confidence among internal stakeholders, enabling participation in collaborative and multi-center initiatives, and attracting growing interest from external academic and industry partners–repositioning it from a passive core to an active institutional asset supporting research quality and sustainability.

Accreditation programs vary in scope, spanning biobanking, clinical and translational laboratories, testing and calibration facilities, quality management systems, and animal research programs [3–6] (Table 1). Despite their differences, they share a common backbone of governance, documentation, training, and continuous improvement. Because accreditation involves independent, third-party verification, it embeds accountability.

**Table 1.** Accreditation and quality frameworks–administrative summary at a glance. Major accreditation/quality programs differ in scope but converge on governance, documentation, training/competency, and continuous improvement. The resulting artifacts create auditable evidence that lowers risk and improves reproducibility across the enterprise

Program	Scope & typical users	Core requirements (plain language)	Evidence produced	Typical inspection cadence*
CAP biorepository	Human biospecimens; academic/health system biobanks	Consent alignment; preanalytical controls; storage and monitoring; chain of custody; distribution traceability	SOPs; training/competency; freezer monitoring and excursion logs; specimen QC and custody records; LIMS audit trails	Biennial external inspection
CAP/CLIA	Labs generating data for clinical care; some translational cores	Assay validation; proficiency testing; QC programs; documentation & corrective actions	Validation reports; QC charts; PT results; CAPA records; staff competency	Biennial (CAP)/biennial (CLIA)
ISO 20387 (Biobanking)	Biobanks of human/animal/other materials; research and clinical interfaces	Quality management; sample lifecycle control; staff competence; method suitability	Quality manual; controlled SOPs; training matrix; sample lifecycle records; performance metrics	3-year certification with annual surveillance
ISO/IEC 17025	Testing and calibration labs (e.g., instruments/assays)	Method validation; measurement traceability; uncertainty; equipment calibration/maintenance	Validation/verification files; calibration certificates; uncertainty budgets; equipment logs	3-year certification with annual surveillance
ISO 9001	Any org seeking a general QMS framework	Process control; risk-based thinking; document control; continual improvement	Quality policy/targets; process maps; internal audit and management review records	3-year certification with annual surveillance
AAALAC international	Animal care and use programs	Standards-based animal welfare; veterinary care; facilities and training	Program description; site-visit reports; corrective actions; training documentation	Triennial site visit with annual reporting

**Figure 1.**
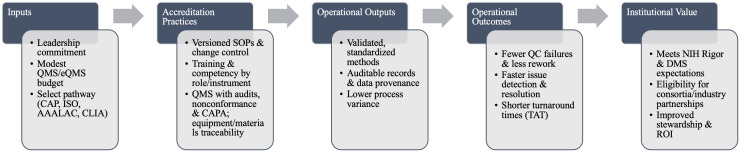
Accreditation logic model for core facilities. Inputs (leadership, modest QMS investment, accreditation pathway) drive accreditation practices (versioned SOPs, role-specific competency, QMS/CAPA, and traceability) that produce operational outputs (validated methods, auditable provenance, and lower variance), yielding outcomes (fewer QC failures, faster resolution, shorter TAT) and, ultimately, institutional value (alignment with NIH Rigor & DMS, eligibility for partnerships, improved stewardship/ROI).

Accreditation operationalizes rigor through four core mechanisms: standardized and validated methods; defined training and competency requirements; formal quality management systems with audit and corrective action; and systematic control of equipment and materials. Standards such as ISO/IEC 17025 (testing/calibration), ISO 20387 (biobanking), and CAP/CLIA (clinical labs) codify these requirements and reduce avoidable variability [3–6] (Figure 1). These same elements map directly onto NIH Rigor & Transparency expectations and the NIH Data Management & Sharing Policy, strengthening both grant applications and publications (Supplementary Figure 1).

Accreditation operationalizes rigor through four core mechanisms: standardized and validated methods; defined training and competency requirements; formal quality management systems with audit and corrective action; and systematic control of equipment and materials. Standards such as ISO/IEC 17025 (testing/calibration), ISO 20387 (biobanking), and CAP/CLIA (clinical labs) codify these requirements and reduce avoidable variability [3–6] (Figure 1). These same elements map directly onto NIH Rigor & Transparency expectations and the NIH Data Management & Sharing Policy, strengthening both grant applications and publications (Supplementary Figure 1).

Beyond quality and rigor, accreditation delivers strategic and financial value. As budgets tighten, funders and industry partners demand accountability and operational reliability; accreditation signals readiness for participation in initiatives requiring formal quality frameworks, including the NIH *All of Us* Research Program and the European BBMRI-ERIC network [7,8]. Accredited service centers can engage immediately, whereas non-accredited facilities may face barriers to entry. Accreditation also strengthens institutional grant competitiveness. Shared resources are central to NIH center mechanisms such as P30, P50, and U54 awards, where reviewers scrutinize governance, quality control, and sustainability; accreditation provides external validation of these elements. Industry collaborators similarly prefer accredited partners to mitigate risks related to data loss, specimen mishandling, or inconsistent results.

Together, these external drivers translate into tangible institutional benefits and improved return on investment. From experience, accreditation has coincided with measurable changes in utilization and sustainability. Over the first four years following initial accreditation, external usership increased more than four-fold, reflecting growing engagement from both academic collaborators and for-profit partners seeking accredited infrastructure. During the same period, internal clinical research studies supported by the center increased by more than 300% in revenue, underscoring the role of accreditation in enabling clinical and translational research. Importantly, these shifts were accompanied by a transition to full cost recovery, eliminating the need for subsidy. While these outcomes cannot be attributed to accreditation alone, they illustrate how accreditation can function as a catalyst for growth.

One of the most overlooked benefits of accreditation is its ability to break down silos. Accreditation rarely affects a single process in isolation; instead, it forces alignment across documentation, governance, training, and data tracking. For example, when preparing for CAP accreditation, a biobank must coordinate with other centers to align specimen handling workflows, with the clinical research office to ensure consent compliance, and with IT to implement a laboratory information management system (LIMS). The result is not just one accredited unit but a ripple effect of standardization across the enterprise. In these ways, accreditation catalyzes efficiency and integration, pushing institutions to adopt common platforms, reduce duplication, and share best practices, all outcomes that extend well beyond compliance.

Accreditation is not without costs–it requires financial investment, dedicated personnel time, and meaningful cultural adaptation. Documentation requirements and audit cycles may initially be perceived as restrictive by staff accustomed to more flexible workflows. Accreditation can also introduce operational rigidity: standardized procedures may slow adoption of novel methods, and audits may feel burdensome. Smaller or highly specialized service centers may find that the return on full accreditation does not justify the expense. These realities underscore the importance of strategic decision-making. Accreditation should be pursued where it aligns with institutional mission, scale, and stakeholder expectations, rather than a one-size-fits-all solution. Importantly, even when full accreditation is not pursued, adoption of its core practices–standardization, training, and audits–can yield measurable improvements in rigor and reproducibility.

Amid increasing scrutiny of research stewardship, accreditation offers a clear signal of accountability. For patients donating biospecimens, it assures that their contributions will be handled with rigor; for funders, it demonstrates that resources will not be wasted on irreproducible or low-quality data; and for the public, it reinforces confidence that institutions are serious about stewardship and transparency. In an era of shrinking budgets and rising scrutiny, accreditation allows service centers to be visible engines of institutional resilience. Research is only as strong as its foundation, and accreditation ensures that foundation is built to last.

## Supporting information

10.1017/cts.2026.10241.sm001 Olson supplementary material Olson supplementary material

## Supplementary material

The supplementary material for this article can be found at https://doi.org/10.1017/cts.2026.10241.
