# Supplementary material for: Beyond compliance: How accreditation can strengthen trust in science and research infrastructure
Source: J Clin Transl Sci. 2026 Jan 21;10(1):e25. doi: 10.1017/cts.2026.10241 (PMC12895486; doi:10.1017/cts.2026.10241)
Supplement: Olson supplementary material [file S2059866126102416sup001.pdf]

Supplementary Figure 1. Alignment of NIH Rigor & Transparency expectations with accreditation artifacts. Checks indicate primary linkages between NIH elements (premise/method validity; rigorous study design; relevant biological variables; authentication of key resources; DMS) and operational evidence produced by accredited cores (validated, versioned methods; training/competency records; QMS audit trails/CAPA; equipment calibration/traceability; resource authentication).

	Validated, versioned methods (SOPs + change control)	Training & competency records (role/instrument)	QMS audit trail & CAPA (audits, deviations)	Equipment calibration & measurement traceability	Resource authentication (RRIDs, COAs, reference materials)
Scientific premise & methodological validity	✓	✓	✓	✓	✓
Rigorous study design (randomization, blinding, sample-size)	✓	✓	✓		
Relevant biological variables (SABV)	✓	✓			✓
Authentication of key resources					✓
Data Management & Sharing (DMS)	✓		✓	✓	✓
